# First experiences in treatment of low-grade glioma grade I and II with proton therapy

**DOI:** 10.1186/1748-717X-7-189

**Published:** 2012-11-09

**Authors:** Henrik Hauswald, Stefan Rieken, Swantje Ecker, Kerstin A Kessel, Klaus Herfarth, Jürgen Debus, Stephanie E Combs

**Affiliations:** 1 Department of Radiation Oncology, University of Heidelberg, INF 400, Heidelberg, 69120, Germany

**Keywords:** Proton therapy, Ion therapy, Particle therapy, Brain tumor, Low-grade glioma, Glioma

## Abstract

**Background:**

To retrospectively assess feasibility and toxicity of proton therapy in patients with low-grade glioma (WHO °I/II).

**Patients and methods:**

Proton beam therapy only administered in 19 patients (median age 29 years; 9 female, 10 male) for low-grade glioma between 2010 and 2011 was reviewed. In 6 cases proton therapy was performed due to tumor progression after biopsy, in 8 cases each due to tumor progression after (partial-) resection, and in 5 cases due to tumor progression after chemotherapy. Median total dose applied was 54 GyE (range, 48,6-54 GyE) in single fractions of median 1.8 GyE. Median clinical target volume was 99 cc (range, 6–463 cc) and treated using median 2 beams (range, 1–2).

**Results:**

Proton therapy was finished as planned in all cases. At end of proton therapy, 13 patients showed focal alopecia, 6 patients reported mild fatigue, one patient with temporal tumor localization concentration deficits and speech errors and one more patient deficits in short-term memory. Four patients did not report any side effects. During follow-up, one patient presented with pseudo-progression showing worsening of general condition and brain edema 1–2 months after last irradiation and restitution after 6 months. In the present MR imaging (median follow-up 5 months; range 0–22 months) 12 patients had stable disease, 2 (1) patients partial (complete) remission, one more patient pseudo-progression (differential diagnosis: tumor progression) 4 weeks after irradiation without having had further follow-up imaging so far, and one patient tumor progression approximately 9 months after irradiation.

**Conclusion:**

Regarding early side effects, mild alopecia was the predominant finding. The rate of alopecia seems to be due to large treatment volumes as well as the anatomical locations of the target volumes and might be avoided by using multiple beams and the gantry in the future. Further evaluations including neuropsychological testing are in preparation.

## Background

In Europe, the estimated annual incidence rate is 4.8 per 100,000 for astrocytic and 0.4 per 100,000 for oligodendroglial central nervous system tumors [1]. Clinical outcome correlates well with the World Health Organization (WHO) classification for brain tumors [2]: with respect to glioma, low-grade glioma (LGG; WHO Grad II) demonstrate a beneficial prognosis compared to WHO Grad III and IV tumors. Therefore, not only local control and survival are important, but also preservation of quality of life (QOL) as well as neurocognitive functioning. Novel radiation modalities, such as proton beam treatments, offer a distinct physical profile enabling a significant reduction of integral dose in the patient. This leads to sparing of normal brain tissue, potentially reducing the risk of neurocognitive sequelae after radiotherapy. Since available clinical data is still limited, this analysis was focused on patients treated with a full course of proton beam therapy only for LGG at the Heidelberg Ion Therapy Center to evaluate treatment feasibility and toxicity.

## Patients and methods

### Patient characteristics

Between 2010 and 2011, 19 patients (median age 29 years; range 4–56 years; 9 female, 10 male; 8 patients <18 years) with LGG (WHO °I/II) were treated with proton therapy at the Heidelberg Ion Therapy Center at the University Hospital of Heidelberg. Three patients suffered from optical pathway glioma. One of the pediatric patients was participating in the HIT-LGG trial, two in the SIOP-LGG trial and one further pediatric patient had previous therapy based on the SIOP-LGG trial. Proton therapy was initiated due to tumor progression after initial surgical resection in 8 patients, biopsy in 6 patients and chemotherapy with mainly temozolomide in 5 patients. The median time interval between initial diagnosis and proton beam therapy was 38 months (range 3–132 months). Patients’ characteristics are found in Table 1.

**Table 1:** Overview on patients' characteristics and treatment

**Patient**	**Gender**	**Age**	**Histology**	**Localization**	**Optic pathway glioma**	**CTV [ccm]**	**Beam count**	**Isodose level in scalp [%]**	**Previous therapy**	**Total dose (GyE)**	**Single dose (GyE)**	**Time interval between inital diagnosis and initiation of proton beam therapy [months]**
**1**	male	10	pilocytic astrocytoma WHO °I	central	no	11	2	10	resection	54	2	45
**2**	male	38	pilocytic astrocytoma WHO °I	central	no	35	2	30	resection	54	1,8	20
**3**	female	12	pilomyxoid astrocytoma WHO °II	central	yes	48	2	30	chemotherapy	54	1,8	>116
**4**	male	37	pilocytic astrocytoma WHO °I	central	no	220	2	30	resection	54	2	44
**5**	female	17	pilocytic astrocytoma WHO °I	central to peripheral	no	99	2	70	resection	54	1,8	80
**6**	male	39	astrocytoma WHO °II	central to peripheral	no	266	2	70	chemotherapy	54	1,8	69
**7**	female	12	fibrillary astrocytoma WHO °II	central to peripheral	no	212	2	90	resection	48,6	1,8	48
**8**	female	48	astrocytoma WHO °II	central to peripheral	no	75	1	70	biopsy only	54	2	3
**9**	female	36	fibrillary astrocytoma WHO °II	central to peripheral	no	212	2	70	resection	54	1,8	85
**10**	female	38	astrocytoma WHO °II	peripheral	no	187	2	70	biopsy only	54	1,8	11
**11**	female	28	astrocytoma WHO °II	central to peripheral	no	371	2	70	resection	54	1,8	26
**12**	male	56	astrocytoma WHO °II	central to peripheral	no	463	1	70	biopsy only	50,4	1,8	15
**13**	female	36	oligoastrozytoma WHO °II	central to peripheral	no	298	2	70	chemotherapy	54	1,8	42
**14**	male	13	pilocytic astrocytoma WHO °I	central	yes	204	2	30	chemotherapy	54	1,8	132
**15**	male	16	pilocytic astrocytoma WHO °I	central	no	17	2	30	biopsy only	54	1,8	99
**16**	male	29	pilocytic astrocytoma WHO °I	central	no	15	2	10	biopsy only	54	2	4
**17**	male	4	pilocytic astrocytoma WHO °I	central	no	6	2	30	resection	54	1,8	8
**18**	male	7	pilocytic astrocytoma WHO °I	central	yes	19	2	10	chemotherapy	50,4	1,8	33
**19**	female	43	astrocytoma WHO °II	central	no	57	2	30	biopsy only	52,2	1,8	4

### Treatment planning

**Figure 1:**
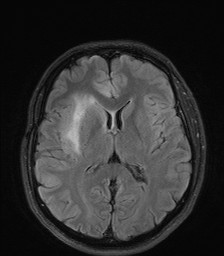
MR-imaging (T2 Flair sequence, not contrast-enhanced).

**Figure 2:**
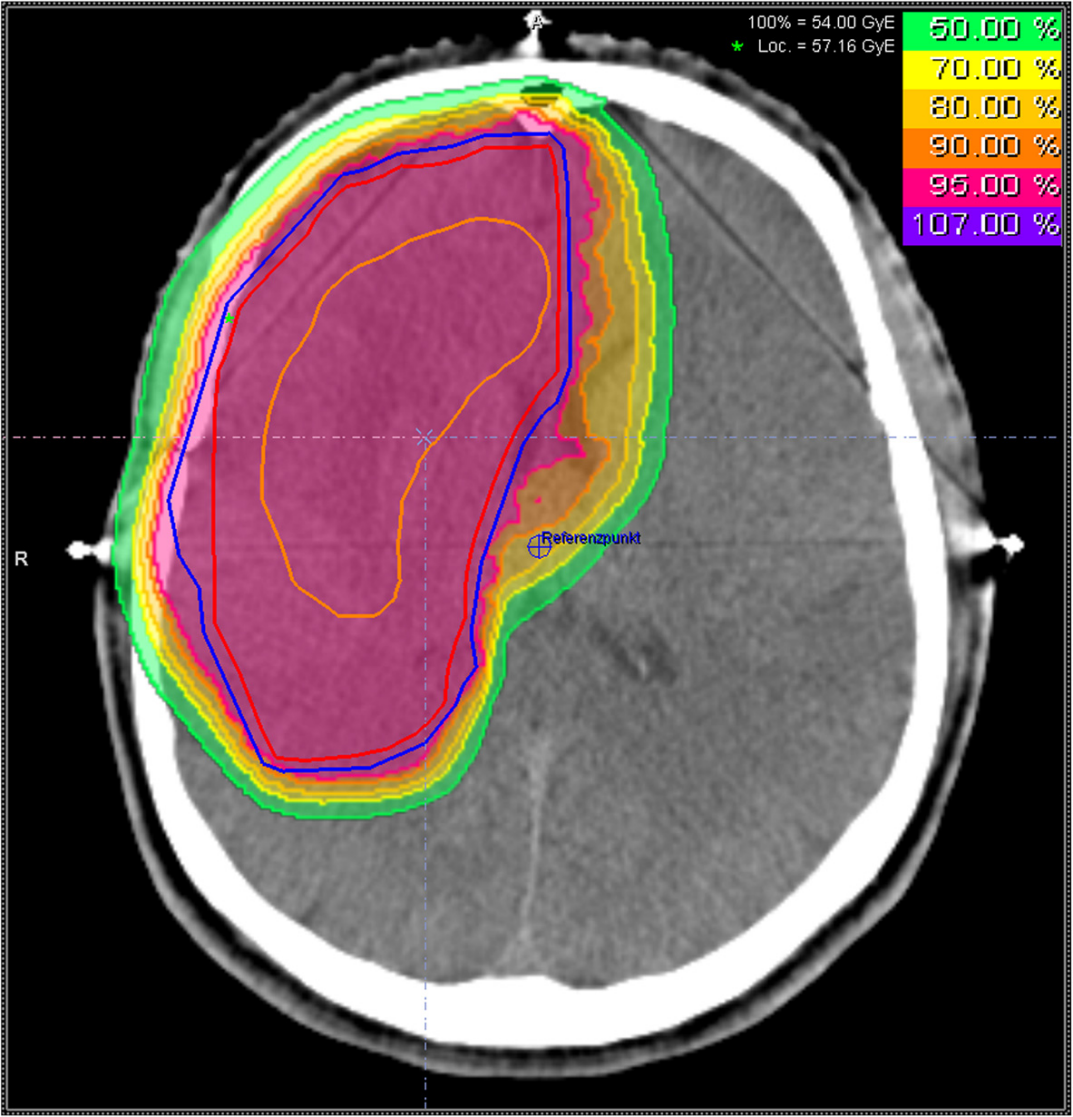
Treatment plan for proton beam therapy including isodose distribution; the orange structure is the GTV, the red structure the CTV and the blue structure the PTV.

The treatment planning was CT- and MRI-based in all cases; eventually additional aminoacid-based positron emission tomography was co-registered for target volume delineation or definition of high-risk regions. Adequate positioning was achieved by individual head masks. The target volume concept included delineation of the gross tumor volume (GTV; defined as the T2-hyperintensity as well as PET-positive regions) adding a 1–2 cm safety margins for the clinical target volume (CTV) to include potential microscopic tumor spread. The planning target volume (PTV) for proton beam therapy included a margin of 3 mm. The prescribed dose was defined as the median dose to the PTV. Furthermore, the PTV was encompassed within the 95-107% isodose level of the prescribed dose. Treatment planning system was syngo.via by the Siemens AG, Germany. See Figures 1 and 2 for a treatment planning example, co-registered is a T2-FLAIR MR-image.

### Particle therapy

Proton beam therapy delivered a median total dose of 54 GyE (range 48.6-54 GyE) in single fractions of median 1.8 GyE (range 1.8-2 GyE) 5–6 times a week to the PTV and was performed using a fixed beam line with non-coplanar beams in active raster-scanning technique with energies between 48.12 MeV/u and 221.06 MeV/u at the Heidelberg Ion Therapy Center. The dose concept for LGG in our institution is 45–54 GyE in 1.8-2 GyE per fraction, so the dose range of 48.6-54 GyE was due to individual factors of the patients like young age or the anatomical localization and tumor volume. Proton beam therapy only was used and not combined with any kind of photon or heavy-ion beam technique or chemotherapy. Patient position was verified prior to each single fraction with orthogonal in-room x-ray imaging. An overview on the treatment is found in Table 1.

### Follow-up and statistics

The first follow-up examination including a MRI of the brain as well as consultation was performed 6–8 weeks after proton beam therapy and every 2–3 months afterwards. Neuropsychological tests or perimetric test of the visual field were not performed on a regular base. If applicable orientating visual filed tests were performed. Tumor response was described by the modified MacDonald/RANO Criteria [3]. All time estimates began with the initiation of proton therapy. Documented side effects were classified according to CTC AE Version 4. Approval of the ethics committee Heidelberg was obtained.

## Results

### Response to treatment

**Figure 3:**
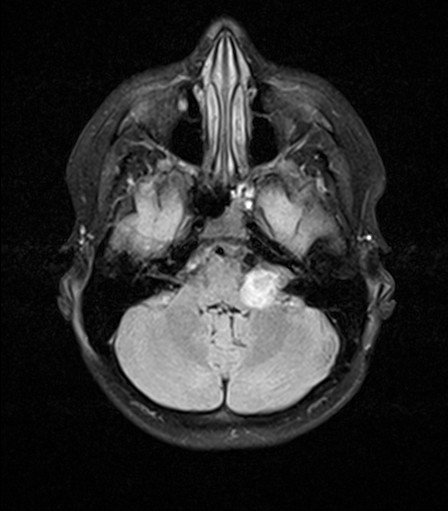
MR-imaging (T2 Flair sequence, not contrast-enhanced) before initiation of proton beam therapy in a juvenile patient with LGG.

**Figure 4:**
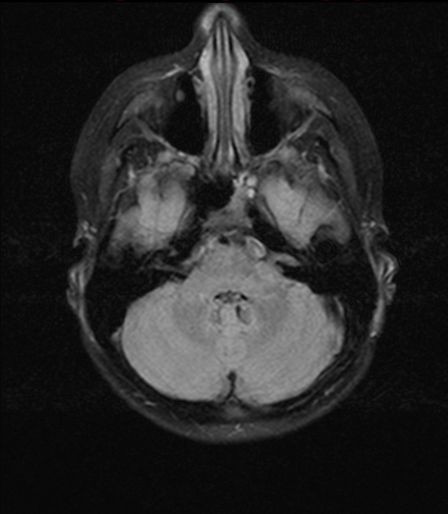
MR-imaging (T2 Flair sequence, not contrast-enhanced) during follow-up after proton beam therapy in a juvenile patient with LGG.

Proton therapy was finished as initially proposed in all patients. In the latest MRI examinations which were performed after a median follow-up time of 5 months (range, 0–22 months), 12 patients had stable disease and 2 (1) patients partial (complete) remission. The follow-up imaging in one juvenile patient with LGG is seen in Figures 3 and 4. During the follow-up visits, two patients were diagnosed with pseudo-progression. One of these two patients presented with pseudo-progression in MRI showing worsening of general condition and brain edema approximately 1–2 months after the last treatment day and restitution after 6 months. The other patient was diagnosed with most likely pseudo-progression 4 weeks after finishing proton therapy based on the clinical course and the typical MRI changes, even though the patient did not have further follow-up imaging so far and tumor progression is a valid differential diagnosis. Furthermore, one patient had to be diagnosed with tumor progression approximately 9 months after finishing proton treatment.

### Side effects

At the end of proton therapy, in 4 patients no acute side effects were observed. Thirteen patients showed focal alopecia CTC °II within the proton beam portal of entry. Radiation induced dermatitis of moderate or higher degree was not documented. Six patients reported mild fatigue. One patient with a tumor localization in the temporal lobe reported deficits in concentration and speech errors (CTC °I-II) and one other patient deficits in short-term memory (CTC °I-II). The documented maximal acute side effects were seen in Table 2. However, no formal testing of neurocognitive functioning was performed. In two patients with optical pathway glioma, tumor-related pre-existing unilateral amaurosis was documented. Additionally, one of these two patients had a contralateral hemianopsia. Treatment related visual field changes were not documented.

**Table 2:** Maximal acute side effects in patients treated with proton beam therapy for low-grade glioma

**Type of side effect**	**n**
**Alopecia CTC °II**	13
**Fatigue CTC °I-II**	6
**Deficits in concentration CTC °I-II**	1
**Deficits in speech CTC °I-II**	1
**Deficits in short term memory CTC °I-II**	1

## Discussion

This analysis reports on outcome of 19 patients treated between 2010 and 2011 with proton beam therapy for LGG at the Heidelberg Ion Therapy Center. Proton beam therapy was applied to a median total dose of 54 GyE in single fractions of median 1.8GyE 5–6 times a week. Treatment was tolerated without high-grade side effects. Acute toxicity of the raster-scanning technique for these low-grade tumors is low. The patients will be followed prospectively. Due to the relatively long survival in patients with LGG, long-term side effects as impaired neurocognitive function become relevant. This issue was addressed by Brown et al. in a review on neurocognitive effects of radiotherapy in LGG [4]. The authors concluded that by modern treatment techniques as MRI-based target volume delineation and the use of multiple overlapping conformal beams, the impact on neurocognitive function could be minimized. So, high conformal and normal brain tissue sparing radiation techniques provide us the potential to further minimize the risk of late neurocogintive impairment. A treatment option with a high potential for normal tissue sparing is e. g. proton beam therapy. Recently Beltran and his team reported on 14 pediatric patients with craniopharyngioma treated with 54 Gy photons. On this base different alternative treatment plans –including intensity-modulated radiotherapy, intensity-modulated proton beam therapy and double-scatter proton beam therapy- were calculated [5]. The radiation dose to the whole-brain as well as –body was significantly decreased by proton beam therapy compared to intensity-modulated radiotherapy. Best treatment plans concerning conformal target coverage and sparing of normal tissue were achieved by intensity-modulated proton beam therapy while being very vulnerable to variations of the target volume on the other hand. Furthermore, not only in children but also adults the risk for secondary cancers after initial radiotherapy becomes crucial. A treatment plan comparison by Athar and Paganetti showed that scanned proton beam therapy had the lowest risk for out-of-field secondary cancers during lifetime compared to passive scattered proton therapy or intensity-modulated radiotherapy [6]. Furthermore, the risk for developing secondary cancers after irradiation of the brain was retrospectively analyzed in 370 children with a median age of 8.1 years by Galloway et al.: In total, 18 secondary cancers were diagnosed in 16 patients median 18.9 years after the associated radiation therapy [7]. The median time to diagnosis of secondary meningioma (n=10) was 22 years, while secondary glioma (n=4) were diagnosed after median 15 years. The incidence rates for secondary cancers at 10, 20 and 30 years after the associated irradiation in childhood were 3%, 8% and 24%, respectively. In conclusion, it seems justified to us, to perform radiation treatments especially in -but not only- children and young adults, if possible as a proton beam therapy, optimally with scanned protons. Clinical data on particle therapy in LGG is rare. In 2002 the colleagues from Loma Linda reported on the first 27 pediatric patients treated with proton beam therapy for LGG [8]. The mean age at time of treatment was 8.7 years and treatment doses between 50.4 GyE and 63.0 GyE in single fractions of 1.8 GyE. The mean follow-up time was 3.3 years and 6 patents were reported to have in-field tumor recurrence. All children with local tumor control maintained their performance status. So the authors concluded that proton beam therapy is safe and efficacious, especially in situations, when high dose conformity is warranted due to central tumors or close relation to the optic pathway. The colleagues from Chiba recently published the results on 14 patients treated between 1994 and 2002 with carbon ions for WHO °II diffuse astrocytoma in a dose-escalation study [9]. The patients were treated with 50.4 to 55.2 GyE carbon ions and for analysis divided into 2 groups according to the treatment dose. Patients in the high-dose group (55.2 GyE, n=5) showed a significantly longer median progression-free survival (91 months vs. 18 months) as well as median overall survival compared to the low-dose group (46.2-50.4 GyE, n=9). The median overall survival for all Patients was 53.4 months and the 5- and 10-year overall survival rates were 43% and 36%, respectively. Acute side effects were minor, while 2 patients of each group had MR-imaging based LENT-SOMA grade 3 late reactions of the brain. These grade 3 late reactions regressed ether spontaneously or after short course of steroids.

Considering MRI-based follow-up examinations, the differentiation of treatment related effects (including radiation necrosis) and tumor progression might be problematic. The rate of radiation induced brain necrosis in glioma patients treated with radiotherapy alone is up to 3.4%, while radiation induced brain necrosis seemed to be unlikely in radiation doses below 50 Gy in 25 fractions but increased with addition of concurrent chemotherapy [10]. Meyzer et al. reported on a n asymptomatic 14 years old boy presenting with new contrast enhancement 6 months after proton therapy (54 GyE) for a low-grade oligodendroglioma located in the tectal region [11]. The colleagues performed a dynamic susceptibility contrast MR-imaging as previously described for differentiation between post-treatment changes and high-grade glioma recurrence by Hu et al. [12]. Meyzer et al. favoured the hypothesis of pseudo-progression, which was treated with weight-adapted oral steroids for one month. Follow-up MR-imaging showed gradual improvement and finally complete resolution of these changes in MR-imaging. Furthermore, MR-spectroscopy can help in differentiation between tumor recurrence and radiogenic changes [13]. However, recently the colleagues from the German Cancer Research Center compared dynamic susceptibility-weighted contrast-enhanced (DSC), dynamic contrast-enhanced (DCE), and proton spectroscopic imaging ((1)H-MRSI) for treatment monitoring and concluded that DSC MR-imaging would be best for identification of tumor progression in glioma [14]. Pseudo-progression occurred in our ladder in 11% of patients. Still, pseudo-progression is a challenging problem during follow-up examinations and could cause emotional stress not only in the affected patients. Radiobiological effects on the skin were reviewed for example by Malkinson et al. in 1981 and described as injure to the cutaneous vasculature and germinative cells causing for example reproductive failure [15]. Radiogenic effects in the hair matrix cells could cause alopecia, which might be permanently in dosages of 7–8 Gy or more. The relatively high rate of alopecia within the proton beam portal of entry in our cohort seems to be related to the anatomical situation and the localization of the target volumes with isodose levels of up to 90% within the scalp. In an upcoming study at our institution, the treatment of patients with LGG will be evaluated prospectively including neuropsychological testing to further improve the knowledge on proton beam therapy in LGG and its effects on quality of life and neuropsychological function.

## Conclusion

Regarding early side effects, mild alopecia was the predominant finding. The rate of alopecia seems due to large treatment volumes as well as the anatomical locations of the target volumes and might be avoided by using multiple beams as well as the gantry in the future. Further evaluations within a prospective clinical trial, including neuropsychological assessment, are in preparation.

## Competing interests

No competing interests do exist.

## Authors’ contributions

HH: analysis and interpretation of data, writing manuscript SR: critically revision for important intellectual content, interpretation of data SE: acquisition and analysis of data KK: acquisition and analysis of data KH: critically revision for important intellectual content, interpretation of data JD: critically revision for important intellectual content, interpretation of data SEC: substantial contributions to conception and design; critically revision for important intellectual content; final approval for publication. All authors have read and approved the final manuscript.
